# Micro-Particle Operations Using Asymmetric Traps

**DOI:** 10.1038/s41598-018-37454-1

**Published:** 2019-02-04

**Authors:** Jaesung Lee, Sarah E. Mena, Mark A. Burns

**Affiliations:** 1 Department of Chemical Engineering, University of Michigan, Ann Arbor, 48109 USA; 2 Department of Biomedical Engineering, University of Michigan, Ann Arbor, 48109 USA

## Abstract

Micro-particle operations in many lab-on-a-chip devices require active-type techniques that are accompanied by complex fabrication and operation. The present study describes an alternative method using a passive microfluidic scheme that allows for simpler operation and, therefore, potentially less expensive devices. We present three practical micro-particle operations using our previously developed passive mechanical trap, the asymmetric trap, in a non-acoustic oscillatory flow field. First, we demonstrate size-based segregation of both binary and ternary micro-particle mixtures using size-dependent trap-particle interactions to induce different transport speeds for each particle type. The degree of segregation, yield, and purity of the binary segregations are 0.97 ± 0.02, 0.96 ± 0.06, and 0.95 ± 0.05, respectively. Next, we perform a solution exchange by displacing particles from one solution into another in a trap array. Lastly, we focus and split groups of micro-particles by exploiting the transport polarity of asymmetric traps. These operations can be implemented in any closed fluidic circuit containing asymmetric traps using non-acoustic oscillatory flow, and they open new opportunities to flexibly control micro-particles in integrated lab-on-a-chip platforms with minimal external equipment.

## Introduction

Microfluidic applications of micro-particles require precise particle operations including separation, sorting, focusing, and solution exchange^1–8^. To accomplish such operations, micro-particles are controlled using either active or passive techniques. Active techniques rely on migration of micro-particles subjected to external force fields including magnetic^9–15^, electric^16–23^, and acoustic^24–29^ fields. These active techniques have advantages such as dynamic operation and flexible tuning of the operation conditions with no change in their physical dimensions. Unlike active techniques, passive techniques rely solely on hydrodynamics or mechanical interaction between particles and physical obstacles. The absence of external force fields makes passive techniques suitable for applications in low resource environments.

For passive techniques, a range of practical functions^30–40^ and unprecedented applications such as rare cell isolation^41–43^ and mechanical characterization of single cells^44–48^ have been demonstrated in the format of continuous flow. However, this type of flow limits the number of independently controllable fluids^49,50^, allows for potential cross-contamination^51^, and presents challenges in metering and aliquoting reagents. Such restraints can be avoided by developing a passive technique relying on non-continuous flow. For that purpose, we previously designed the “asymmetric trap”, a unique mechanical trap, for which interactions with particles vary with flow-direction across the trap in non-acoustic oscillatory flow^52,53^. The array of asymmetric traps under the oscillatory flow field is capable of inducing particle displacement with no net transport of the liquid inside a closed fluidic chamber. The particle dynamics in asymmetric traps are predictable based on the physical dimensions of the trap and the particle.

The objective of the present paper is to develop three practical particle operations using asymmetric traps: size-based segregation, solution exchange, and focusing/splitting of micro-particles. For particle segregation, up to three types of particles were separated according to their size in tens of fluid oscillations (oscillation frequency = 80–250 ms). Their separation was possible due to the size-dependent character of particle dynamics in asymmetric traps. The second operation transported micro-particles between two solutions, as would be required in buffer-exchange operations. Finally, focusing and splitting of particles into desired areas of the fluidic chamber were achieved by capitalizing on the transport polarity of the array. Design considerations are given for each of the applications including the minimum number of trap rows required and the optimal amplitude and number of fluid oscillations. The size-dependency of the particle dynamics, transport polarity, and multiplexing capability can be combined to create diverse functions beyond the particle operations demonstrated in this report.

## Results and Discussion

### Asymmetric traps and one-way particle transport

**Figure 1:**
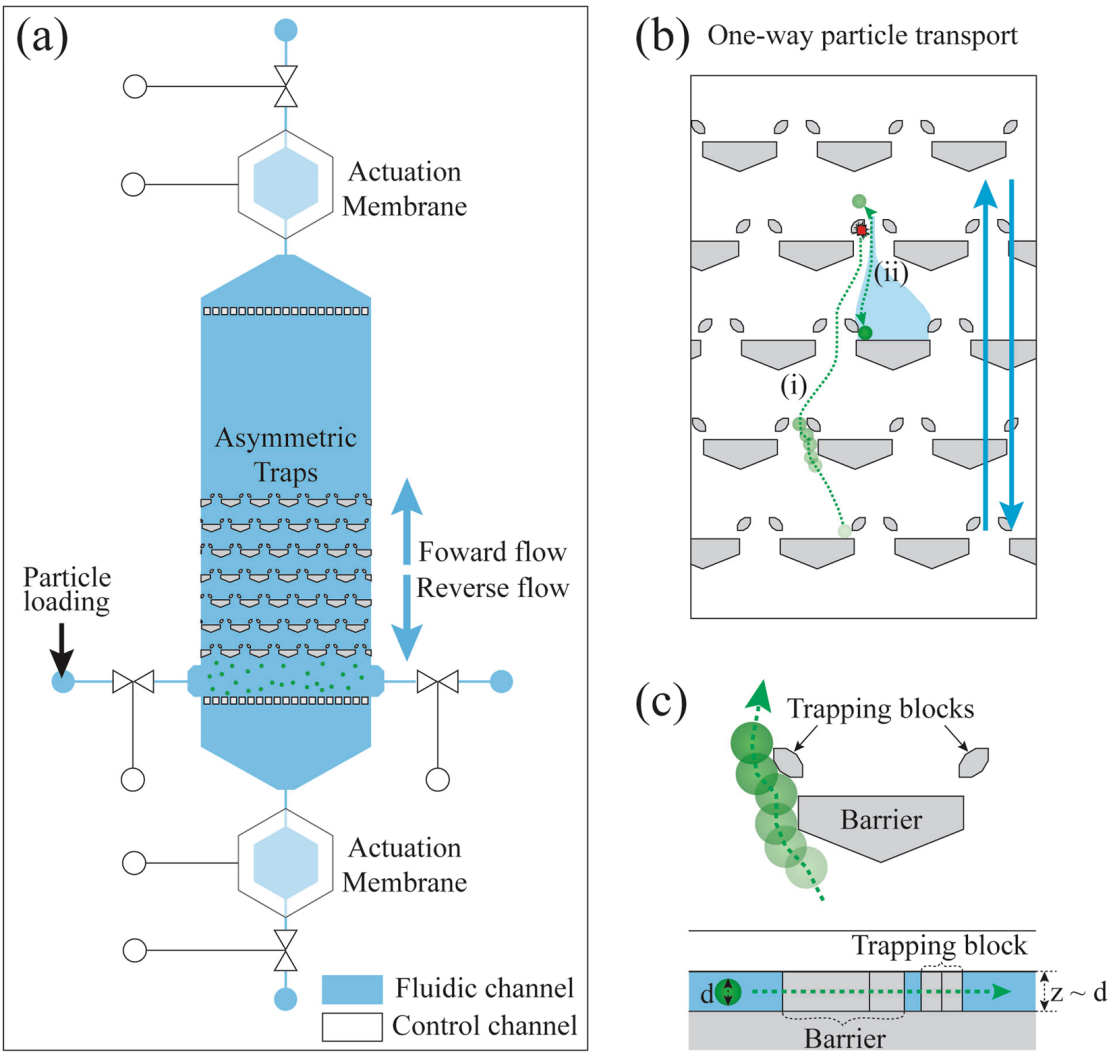
Diagrams of the device and one-way particle transport. (**a**) Diagram of the device. An oscillatory flow field across asymmetric traps is made by pneumatically pressing two actuation membranes in turn. (**b**) The steps of one-way particle transport: (i) During forward flow, the particle travels around the traps and bumps into trapping blocks. The bumping during forward transport causes the particles to shift into a different streamline. (ii) During reverse flow, the micro-particle, having been laterally shifted into the capturing stream, is mechanically captured in the gap between the barrier and a trapping block. The blue arrows represent fluid flows. (**c**) In the regime of one-way particle transport, a particle in forward flow avoids capturing due to lateral shift caused by the barrier of asymmetric trap. It is worthwhile to note that the channel height is comparable to particle diameter (Top: overhead view; Bottom: side view).

When micro-particles interact with asymmetric traps in an oscillatory flow field, their behavior can be classified as one of five types, as described in our previous study: one-way particle transport, symmetric passage, symmetric capturing, trap skipping in zig-zag mode, and trap skipping in bump mode (Supplementary Note S1, Figs S1 and S2)^53^. In the present work, we selected one-way particle transport as the base mode to accomplish the operations of segregation, solution exchange, and focusing and splitting of the particles. One-way particle transport is driven by an oscillatory flow field across asymmetric traps. The oscillatory flow is made by cyclically pressing two actuation membranes located on both ends of the fluidic chamber (Fig. 1a). One-way particle transport consists of a series of actions that produce a net positive displacement of a particle in oscillatory fluid flow. During forward flow, the particles travel around the traps and bump into trapping blocks ((i) of Fig. 1b). An event of bumping during forward transport (Fig. 1c) causes the particles to shift into a different streamline. Then, during reverse flow, the micro-particles, having been laterally shifted into a capturing stream, are mechanically captured in the gap between the barrier and a trapping block ((ii) of Fig. 1b). This ratchet-like motion of the particles during a fluid oscillation results in a net positive displacement of the particles in one direction while the fluid has no net displacement.

**Figure 2:**
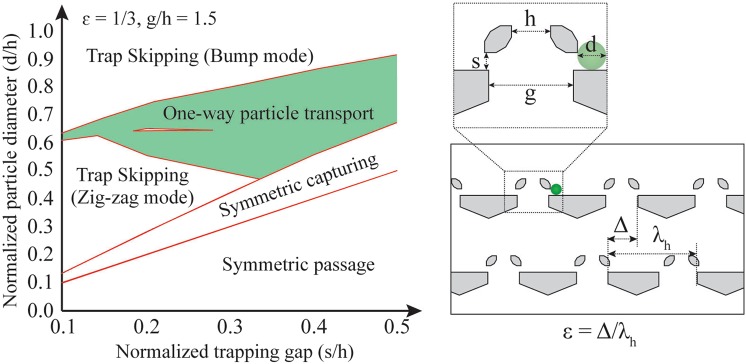
Phase diagram for particle motion in asymmetric arrays. This phase diagram was theoretically obtained and experimentally validated^53^. The region for one-way particle transport (shaded in green) is shown for a row shift fraction of 1/3 and *g*/*h* of 1.5. Outside of the region for one-way particle transport, other trap-particle interaction regimes occur, having oscillating, non-transport motion of the particles (Supplementary Note S1, Figs S2 and S3). Note that the variables for the geometric dimensions of the row shift fraction, *ε*, of the array of asymmetric traps, particle diameter, *d*, and three gaps (*s*, *g*, and *h*) are shown in the diagram.

Different modes of particle transport can be achieved by adjusting the physical dimensions of the asymmetric traps based on the phase diagrams of particle dynamics (Fig. 2). The phase diagram displays the effect of the particle diameter (*d*), the dimension of the gaps in the array (*s*, *g*, and *h*), and the row shift fraction (*ε*) on the particle dynamics (Figs 1c, S2 and S3)^53^. With this diagram, users can achieve not only one-way particle transport but also other possible particle dynamics (Supplementary Note S1). This controllability of particle dynamics is critical for particle operations using asymmetric traps.

**Figure 3:**
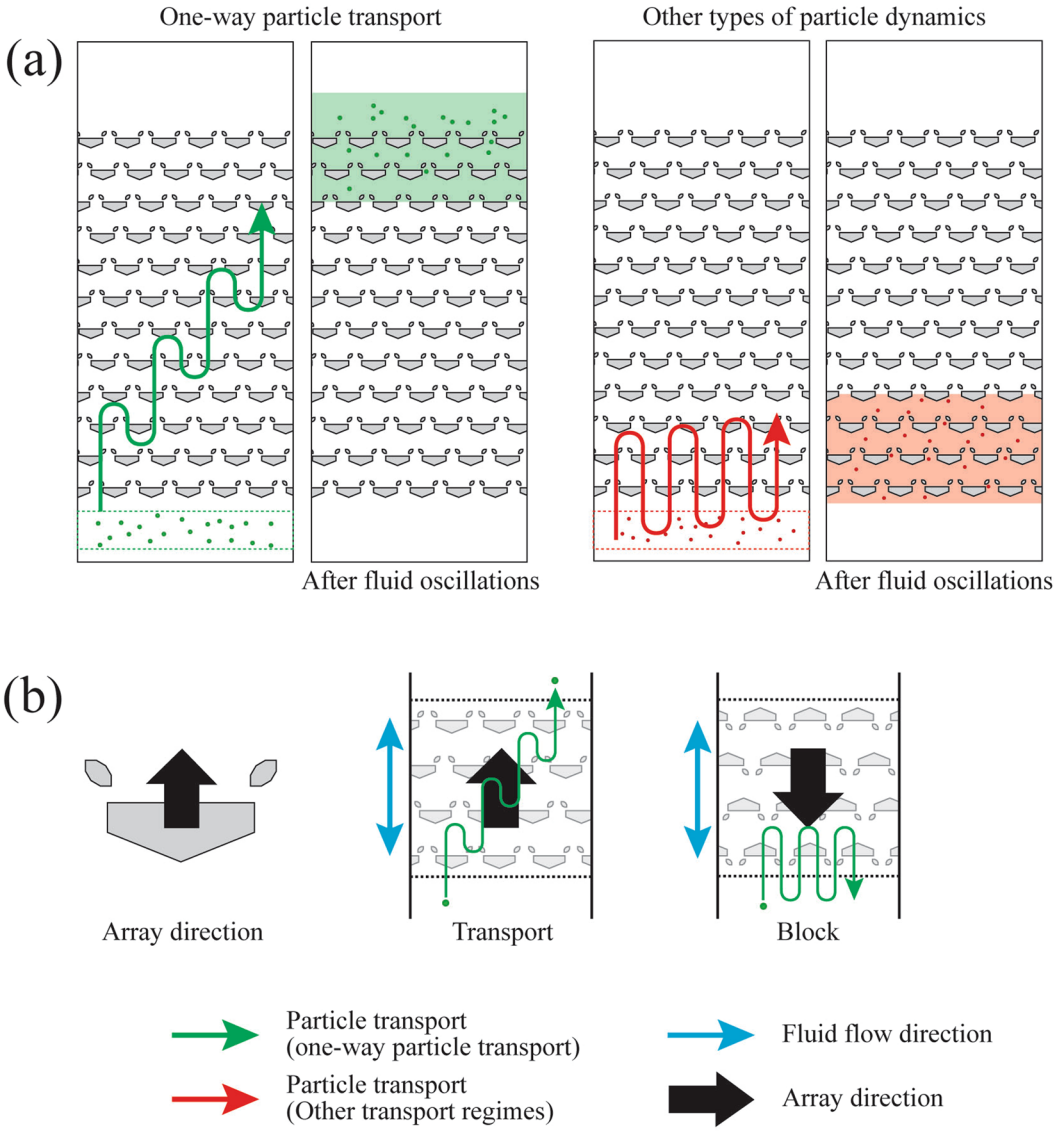
Two features of the one-way particle transport. (**a**) The speed and distance of one-way transport (left, green particles) is much greater than the particle transport of other interaction regimes (right, red particles). It should be noted that the oscillating green and red arrows represent the average vertical movement of particles in the oscillatory flow. The particles do not penetrate through the traps. (**b**) Transport polarity of the asymmetric trap. In the regime of one-way transport, the particle can be transported in only the array direction (shown as black arrows), the direction from the barrier to the trapping blocks. Particle transport in the opposite direction is blocked by the asymmetric traps. The blue arrows represent the direction of fluid flow.

One-way particle transport was selected as the base transport mode for the present study due to two features: (1) the higher transport efficiency compared to the other particle transport modes and (2) the unique transport polarity (i.e., transport in only one direction). In one-way particle transport, the particle moves along the axis of the fluid oscillation by a distance approximately equal to the oscillation amplitude (Fig. 3a). On the other hand, the other transport modes show slower transport or no appreciable displacement of particles during the fluid oscillation^53^. Additionally, net displacement of particles in one-way particle transport occurs in the direction from the barrier towards the trapping blocks (Fig. 3b). This diode-like transport adds flexibility to particle operations, and complex particle operations can be performed by adjusting the orientation of the trapping systems in the arrays.

The following sections show how one-way particle transport can be used, alone or in combination with other modes of particle transport, to control the position and movement of micro-particles in these arrays.

### Segregation of micro-particles

**Figure 4:**
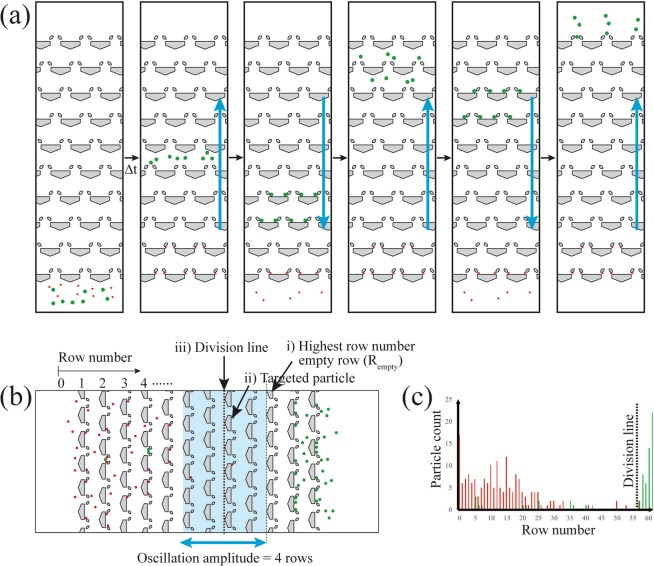
(**a**) Schematic diagram of particle segregation. The target particles (green) exhibiting one-way particle transport are segregated from non-target particles (red) in the symmetric capturing regime. (**b**) The procedure to set the location of the division line in the segregation product. (i) Find a trap row (R_*empty*_) that has the highest row number among the rows void of target particles. (ii) Find target particles in the rows between R_*empty*_ and R_*empty*_- oscillation amplitude (the number of rows the particles move in a single forward oscillation). (iii) In those rows, the division line is drawn at the smallest row number where the targeted particles are present. (**c**) A distribution pattern from a segregation experiment and the location of a division line. Here, the oscillation amplitude is 4 rows.

Segregation of particles in asymmetric traps is possible if one group of particles is in the one-way particle transport regime while another is in the symmetric capturing regime. The particles in the regime of one-way transport (targeted particles) gain a net displacement of approximately the oscillation amplitude during each fluid oscillation. On the other hand, the particles in the regime of symmetric capturing (non-targeted particles) are restricted from advancing in the array because they are trapped during forward and reverse flow (Fig. 4a). Therefore, after a number of fluid oscillations, the targeted particles are transported to the end region of the array while the non-targeted particles remain near their original location (Supplementary Movie S1). Note that the regimes applicable for particle segregation are not limited to one-way transport paired with symmetric capturing. Other combinations of the regimes such as one-way transport vs. symmetric passage or trap skipping can also be used for segregation.

The segregation of a particle binary mixture results in an asymmetric, bimodal distribution across the rows (Fig. 4b). The non-targeted particles in the symmetric capturing regime stay near the entry region of the array (Fig. 4b), while the targeted particles travel to the other end via the one-way transport mechanism. A few of the targeted particles may remain with the non-targeted particle group due to particle size distribution or array clogging. We divide the array after the segregation into two separate regions, a targeted particle-rich area and a non-targeted particle-rich area. We set the location of the division between these two regions at the highest row number row that is void of targeted particles (*R*_*empty*_). The caveat is that, if there are any targeted particle in the rows between *R*_*empty*_ and *R*_*empty*_-oscillation amplitude (the number of rows the particles move in a single forward oscillation), the division line is drawn at the smallest row number where the targeted particles are present. Accounting for the oscillation amplitude when establishing the division line can help differentiate whether the targeted particles outside of the cluster at the exit of the array are the result of a transport failure or from simply being drawn back into the array during reverse flow. In Fig. 4c, the oscillation amplitude is 8, and there are no targeted particles present in the 4 rows immediately before *R*_*empty*_. Therefore, the division line is drawn at row 57. In particular cases where the arrays have a low number of rows or the number of the oscillation is low (<20), there might not be any empty rows between the clusters of particles. In these cases, the division line is drawn at the highest row number where the non-targeted particles are present.

The division line defines two regions: the non-targeted particle region (${N}_{\ast }|{}_{n}$) near the entrance rows of the array, and the targeted particle region (${N}_{\ast }|{}_{t}$) near the end of the array (Fig. 4b). With these definitions, we can evaluate the segregation performance using three expressions: degree of segregation, yield, and purity, as defined below:

1 $${\rm{Degree}}\,{\rm{of}}\,{\rm{segregation}}=\frac{{N}_{n}|{}_{n}+{N}_{t}|{}_{t}}{{N}_{T}}$$

2 $${\rm{Yield}}=\frac{{N}_{t}|{}_{t}}{{N}_{t}}$$

3 $${\rm{Purity}}=\frac{{N}_{t}|{}_{t}}{{N}_{T}|{}_{t}}$$

*N*_*T*_, *N*_*n*_, and *N*_*T*_ are the number of target, non-target, and total particles, respectively. The vertical bar and subscript indicate the location of the particle count (targeted (t) or non-targeted (n) region) (Supplementary Table S2). The degree of segregation shows the ratio of the particles segregated to the total number of the particles. The yield represents the ratio of the targeted particles segregated from the initial mixture. The purity indicates the fraction of targeted particles in the targeted particle region.

**Figure 5:**
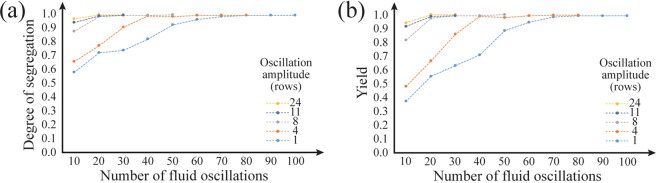
Segregation performance as a function of fluid oscillations at various amplitudes. The number of rows was 60. Every 20 oscillations, a long oscillation of 11 rows was generated to make sure all of the particles enter the array. (**a**) The degree of segregation as a function of the number of the fluid oscillations. As the amplitude of the oscillation increased, a degree of segregation of 1 was more quickly achieved. (**b**) The yield of the segregation as a function of the number of fluid oscillations. Similarly, the number of oscillations required for high yield was smaller for larger amplitudes of oscillation.

Figure 5 shows the effect of the oscillation amplitude and the number of oscillations on the degree of segregation and the yield. It should be noted that an oscillation of 11 rows was applied every 20 oscillations to ensure that the particles located near the entry region entered the array, and this, in turn, induced abnormally high values of the segregation performances for an oscillation of 1 row. For all the tested amplitudes ranging from 1 to 24 rows (60 rows total in the array), the degree of segregation and yield reached high values (>0.96) (n = 3). The data show that smaller oscillation amplitudes require a greater number of oscillations for segregation than larger amplitudes but both conditions reach high values of degree of segregation and yield. Large amplitudes (12 and 24 rows) did not cause undesired long transport of non-targeted particles, which can be attributed to the symmetric capturing interaction between the trap and non-targeted particle. In the regime of symmetric capturing, forward transport of the non-targeted particles are blocked by the asymmetric trap due to particle capturing during forward flow. It should be noted that this experiment had a small number and amplitude of the oscillations (e.g. 20 oscillations, 4 row amplitude) resulting in no empty rows between the targeted and non-targeted particle clusters. For this case, as previously explained, the division line was set at the highest row number where non-targeted particles were present. This location of the division line makes the purity of the segregation always high, so the calculation of the purity of the segregation product for this experiment is not representative.

**Figure 6:**
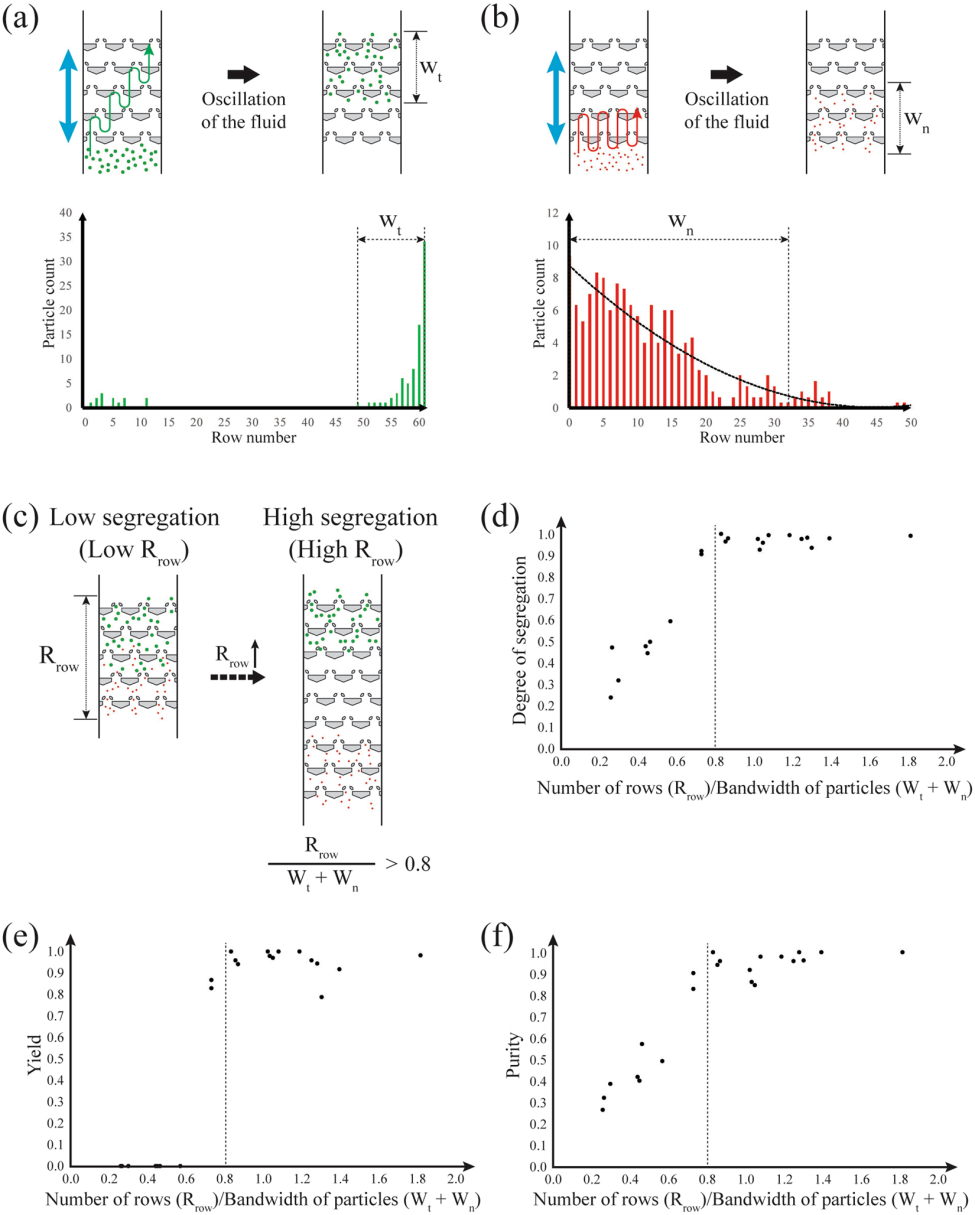
Bandwidth of the particles and its effect on segregation performance. (**a**,**b**) Particle distribution of targeted (**a**) and non-targeted (**b**) particles after fluid oscillations. *W*_*t*_ is the bandwidth of the targeted particles, and *W*_*n*_ is the bandwidth of the non-targeted particles. The bandwidth of the non-targeted particles is defined as the row number where the value of the fitted line becomes less than one. The plot of the non-target particle distribution is the average value of multiple sets of results (n = 3). (**c**) As the ratio of the *R*_*row*_ to *W*_*t*_ + *W*_*p*_ becomes greater than 0.8, all of the segregation performance factors including (**d**) the degree of segregation, (**e**) yield, and (**f**) purity of the segregation reached high values (>0.90).

The number of rows present in an array is a critical parameter affecting the segregation performance. In arrays with a large number of rows, the particles have enough space to concentrate into two distinct regions separated by empty rows, resulting in a higher separation performance. On the other hand, arrays with a small number of rows have a mixed zone of targeted and non-targeted particles in the center that decreases the segregation performance. The number of rows can be non-dimensionalized by the bandwidth, defined as the number of rows where particles are distributed after an experiment. Note that the bandwidth was measured from experiments where targeted and non-targeted particles were tested individually (Figs 6a,b) and is dependent on the number of particles loaded into the array and the number of traps per row, which was fixed at 10 for the segregation experiment. The bandwidth of targeted particles was found to be linearly proportional to the total number of the particles while the bandwidth of non-targeted particles scaled with the square root of the total number of non-targeted particles (Supplementary Fig. S3). As can be seen in Fig. 6c–f, when the ratio between the number of rows and the sum of the individual bandwidths was greater than 0.8, the degree of segregation, yield, and purity remains constant at around 0.9–1.0 (mean ± standard deviation: 0.97 ± 0.02, 0.96 ± 0.06, and 0.95 ± 0.05, respectively). Thus, in order to have a high degree of segregation, the numbers of rows should be higher than 0.8 multiplied by the sum of the bandwidths. The threshold of 0.8 rather than 1.0, as one might anticipate, is attributed to the quadratic fitting for the bandwidth of non-targeted particle (Fig. 6b) and the relatively small number of particles in the overlapping rows compared to other rows of the bandwidth.

**Figure 7:**
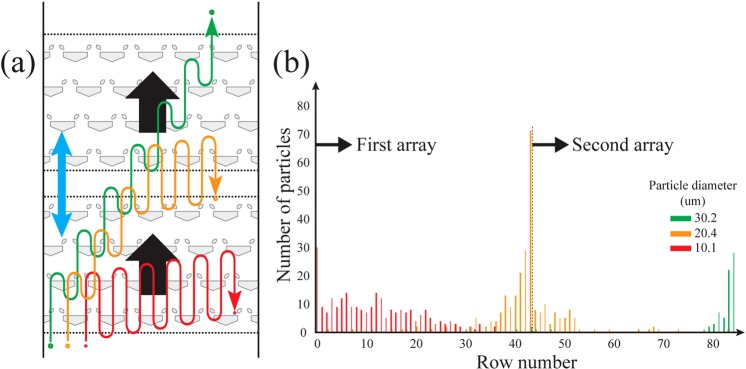
Multiplexed segregation using two arrays in series. (**a**) Schematic diagram of the multiplexed segregation. The first array transports large (green) and medium-sized (yellow) particles. Between those two particle groups, only the large particles are transported by the second array. The small (red) particles stay in the region of the first array. Note that the large displacement arrows show the net direction of vertical motion of the particles. (**b**) A distribution result of particles after segregation. The relative dimension of the first array is *s*/*h* = 0.34, *g*/*h* = 1.5, and *ε* = 1/3, and for the second array is *s*/*h* = 0.50, *g*/*h* = 1.5, and *ε* = 1/2.

Particle segregation was multiplexed by adding a second array with different dimensions in series with the first array (Fig. 7, Supplementary Movie S2). The geometries of the two arrays were designed to segregate a ternary mixture of micro-particles. The first array selectively transported large- and medium-sized micro-particles while preventing transport of the smallest particles. The next array only transported the largest particles (Fig. 7a) while preventing the transport of the medium sized particles. The degree of segregation of the multiplexed segregation was 0.95 ± 0.02 (n = 4). The maximum value of the degree of segregation (~0.97) from the multiplexed segregation was slightly lower than the result of the segregation of the binary mixture (>0.99) described above (Fig. 7b). This difference can be attributed to the interaction regime of the medium and small particles used for the multiplexed segregation. Both of these particles were in the regime of symmetric passage (i.e., the array could not block the forward or reverse movement of the particles during fluid oscillations), and the particles were transported further in the array than when using symmetric capturing^53^.

### Other operations: solution exchange and focusing/splitting of particles

By using the one-way particle transport regime we can exchange the solution surrounding the micro-particles. After sufficient fluidic oscillations, micro-particles are displaced from their original solution, in this case a fluorescent solution, and move into a second solution, a non-fluorescent solution (Supplementary Movie S3). This type of solution exchange is useful to automate surface-bound multi-stage reactions and washing of micro-particles, droplets, and cells in integrated lab-on-a-chip devices^5,8,36,54^. Note that, during the oscillation, some mixing of the solutions occurs. If the speed of the particle transport is not faster than the propagation of the mixing front, the particles cannot be moved to the new solution. The dominant mode of mixing is dispersion as the diffusion coefficient, D, of typical species are at most 10^−5^ cm^2^/s. For 20 oscillations at 300 ms/oscillation or 6s, the resulting diffusion front is on the order of $\sqrt{Dt}$ ~ 100 μm, much shorter than actual displacement of the dispersion front (~5600 μm).

**Figure 8:**
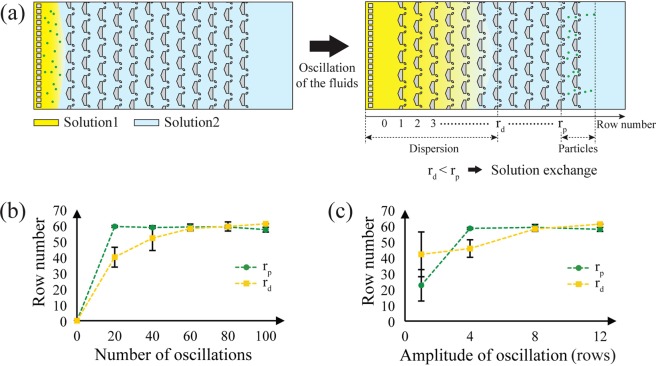
The solution exchange using asymmetric traps. (**a**) Schematic diagram of the solution exchange. The particles were moved from solution 1 to solution 2 by one-way particle transport. The solution exchange occurs if the speed of one-way particle transport is greater than the propagation speed of the dispersion front. (**b**) The effect of the number of fluid oscillations on solution exchange. The amplitude of the fluid oscillation was fixed at 8 rows. (**c**) The effect of the amplitude of the fluid oscillation on solution exchange. The number of fluid oscillations was fixed at 60. The error bars in (**b**,**c**) represent the standard deviation of *r*_*p*_ and *r*_*d*_ measurements (n = 3).

To evaluate the solution exchange at a certain number and amplitude of oscillations, we noted the row number of the dispersion front, i.e. the front of the fluorescent dye dispersion (*r*_*d*_), and the rearmost row where the particles reside (*r*_*p*_) after the oscillations occurred (Fig. 8a). We deem the solution exchange successful if *r*_*p*_ is greater than *r*_*d*_, and we varied the number and the amplitude of the oscillations to observe their influence on the solution exchange. Figure 8b shows an example for a set amplitude of 8 rows. Initially *r*_*p*_ increases much faster than *r*_*d*_ with increasing oscillations, but by oscillation number 60 the two values are indistinguishable, indicating that there is a certain number of oscillations where the solution exchange is possible for a particular length of the array. For the case in Fig. 8b, the solution exchange will be successful for 40 or less oscillations.

We also varied the oscillation amplitude at a set oscillation number of 60, as seen in the graph in Fig. 8c. For amplitudes less than 4 or greater than 8 rows, *r*_*d*_ is larger than or the same as *r*_*p*_ and the solution exchange is not successful. But for amplitudes of 4, *r*_*p*_ is larger than *r*_*d*_ and the solution exchange occurs. This ability to perform solution exchange at oscillation numbers between 20–60 with amplitudes between 4–8 rows, indicates that there is an optimum value for these variables to maximize the ratio of particle transport and minimize the advance of the dispersion front. Besides the amplitude and the number of oscillations, several factors such as the solution concentration, dimensions and density of the asymmetric traps, and Reynolds number of oscillatory flow can vary the speed of the dispersion front. Further studies on these variables can help optimize this phenomenon for particular applications.

**Figure 9:**
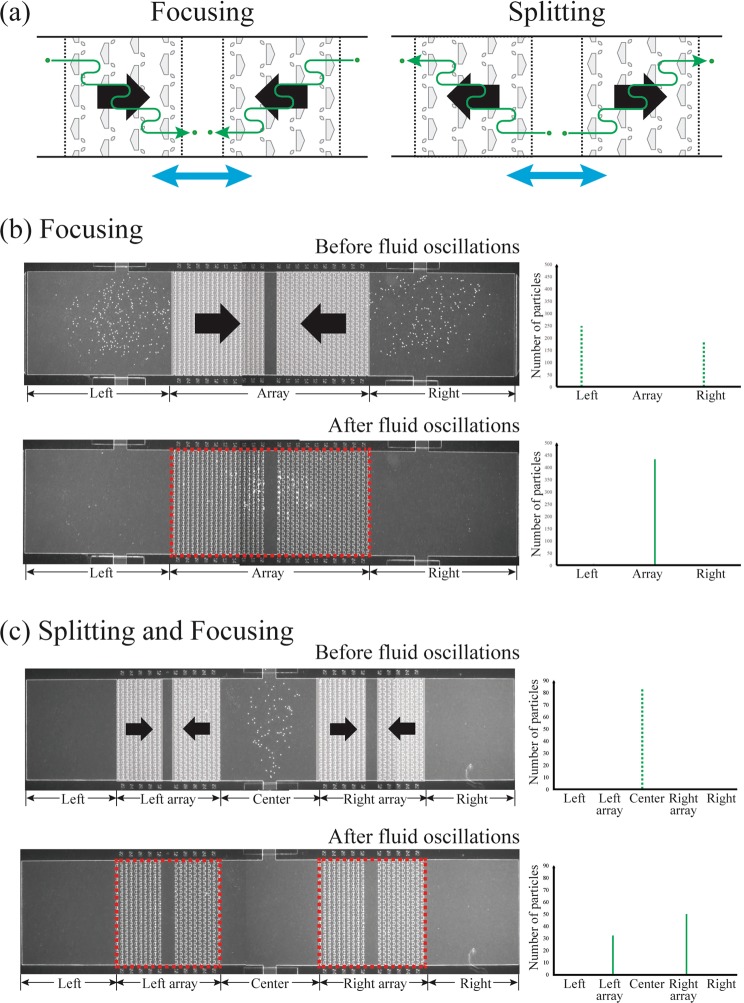
Particle focusing and splitting using asymmetric traps. (**a**) Schematic diagrams of particle focusing and splitting. (9a-left) Arrays pointed towards each other can focus particles in the central area between the arrays. (9a-right) Arrays pointed away from each other can allocate the particles into desired regions. Note that the particles move along only the oscillation direction of the array. (**b**) Experimental result of particle focusing. All the particles loaded are focused into the array area (dashed red boxes) after fluid oscillations (n = 3). (**c**) Experimental result of the particle splitting and focusing. The particles are split and focused into the areas (dashed red boxes) of two coupled arrays after fluid oscillations. The split of the particles was uneven. The ratio of the particles focused in right array to the ones focused in left array was 4.95 ± 3.97 (n = 3).

Next, we used the transport polarity of the asymmetric traps to focus and split micro-particles. Because the transport polarity of the asymmetric traps restricts the direction of transport, multiple arrays having different orientations can enable flexible spatial control of micro-particles. For example, two arrays with opposite polarities (i.e., pointing toward each other) can focus particles in the area between the two arrays (Fig. 9a). On the other hand, two arrays pointing away from each other can split and displace particles outside of the arrays (Fig. 9a). This focusing and splitting can be extended to multiple target regions.

Experimental results for both these cases are shown in Fig. 9b,c. For focusing (n = 3), all the particles were initially loaded at the outer parts of the array (Fig. 9b, time zero) and were focused at the center between the arrays. For splitting (n = 3), the particles were loaded into the center region of a device, and the particles were concentrated at the ends of the arrays (pictures not shown). Finally, a combination of splitting and focusing of particles was achieved by using two coupled arrays (Fig. 9c). All the particles initially injected into the central area were successfully split and focused into two coupled-arrays (n = 3). However, the ratio of particles focused in the right array to the particles focused in the left array was estimated as 4.95 ± 3.97. This uneven split comes from the order of particle transport in oscillatory flow. The particles are transported to the right array at first so the array always captures more particles than left array. Further investigation about capturing probability at the first oscillation will be needed to optimize splitting of particles. Note that the number of particles the array can focus or split is linked to the number of rows in the array. The particles not captured in the array region stay outside of the array. This leaky-character of the asymmetric traps, in turn, can be utilized for sampling the micro-particles during processing.

## Conclusions

In this study, we demonstrated three exemplary operations using asymmetric traps: particle segregation, solution exchange, and focusing and splitting of the particles. Asymmetric traps can be used to isolate one particle type from another using one-way particle transport and symmetric capturing with segregation efficiencies up to 0.99. Additionally, the particles can be transported from one solution to another, accomplishing solution exchange which is particularly important in particle systems that involve binding or reaction at the surface. Finally, using the transport polarity of the asymmetric traps, the particles can be either focused or split by two arrays pointing in opposite directions. These types of particle focusing or splitting can readily deliver micro-particles at multiple locations with a single fluidic operation. Also, the segregation, solution exchange, and focusing/splitting can be conducted at the same time, enabling the possibility of complex functions. Various types of operations other than the ones shown here can be created by multiplexing particle sizes, adjusting the types of trap-particle interactions and transport polarity, and varying the number of arrays.

## Methods

### Device fabrication

The microfluidic devices consist of three layers: a fluidic channel etched in Si, a control channel made of polydimethylsiloxane (PDMS) (Sylgard 184^®^, Dow Corning), and a thin PDMS membrane separating the two channels. A schematic of the device is shown in Fig. 1a. For most experiments, the geometry of the devices is the same as in our previous work^53^ with changes only to the dimensions of the asymmetric traps that were modified for each particle size (Fig. 2 and Table S1). The focusing and splitting experiments used a slightly modified geometry in the loading region of the particles. The structure of the Si fluidic channel was shaped via photolithography and dry etching. A photoresist (SPR 955^TM^-CM, Microchem Corp.) was coated at 3000 rpm on a Si wafer and exposed by a mask aligner (MA/BA6 mask aligner, Karl Suss MicroTec) for 5 s at 30 mW/cm^2^ after a pre-bake for 1 min at 100 °C on a hot plate. The exposed resist was developed (AZ^®^726, MicroChemicals) after a post-bake for 1 min at 110 °C on a hot plate. Deep reactive ion etching (DRIE) (STS Pegasus 4, SPTS Technologies, Ltd.) was applied to the exposed area so that a fluidic channel was etched into the Si surface. The height of the channel was about 40 μm. The control channel of PDMS was cast on a mold made of Si. The pattern on the mold was shaped following the same methods as the Si fluidic channel. Then, the surface of the mold was passivated by a trichlorosilane ((tridecafluoro-1,1,2,2-tetrahydrooctyl)-1-trichlorosilane, UCT) via plasma activation of the surface and vapor phase deposition. The PDMS monomer and cross-linker were mixed (10:1 w/w ratio) and poured in the mold. The PDMS and mold were heated at 80 °C for 1.5 hr in an oven after degassing. The height of the control channel is about 100 μm. The thin PDMS membrane was spin-coated and cured on a Si wafer. The PDMS mixture (10:1 monomer:cross-linker w/w ratio) was spin-coated at 600 rpm and cured at 140 °C for 3 hr on a hot plate. After each part is fabricated, they are aligned and attached via plasma bonding. Both the normally-closed valves and their locations in the fluidic channel were masked to preclude bonding. The detailed dimensions of the traps used are summarized in Table S1.

### Experimental parameters

In the experiments, we controlled four variables: the number and amplitude of particle oscillations, the number of rows, and the number of particles. The number of oscillations was controlled by varying total number of opening and closing events of the solenoid valves in the pressure line. The amplitude of the oscillation was varied with the adjustment of the opening period of the solenoid valves. The relationship between the amplitude and the valve opening time was empirically obtained before the particle operations were conducted. The number of trap rows was varied in the design stage of the device. The number of particles loaded into the array was proportional to the concentration of the particle solution.

### Preparation of micro-particle solutions

Binary mixtures of fluorescent polystyrene particles of 30.2 μm and 20.4 μm diameter (SPHERO^*TM*^ Fluorescent particles, FP-30052-5 and FP-20056-5, Spherotech, Inc.) were used for the segregation experiments. For multiplexed segregation, particles of 10.1, 20.4, and 30.2 μm diameter were used. The focusing and splitting experiments used a solution of 30.2 μm particles. The particles were re-suspended in a density-equilibrated buffer that is a mixed solution of deionized water, OptiPrep^*TM*^ density gradient medium (Sigma-Aldrich Co.), and Tween 20 surfactant of 0.1% v/v (Sigma-Aldrich Co.). The number of the particles in the array was adjusted by varying the concentration of the particles in the suspension. The solution exchange experiments used two solutions including the density-equilibrated buffer and a fluorescein solution (0.004 g/mL, fluorescein sodium salt dissolved in the density equilibrated buffer) (Sigma-Aldrich, Co.). Before the experiment, the particles of 30.2 μm diameter were re-suspended in the fluorescein solution. Once the fluorescein solution was loaded into the device filled with the density-equilibrated buffer, the oscillatory flow displaced the particles from the fluorescein solution to the buffer. The fluorescence of the array region was measured before and after the segregation.

### Image analysis

The images of the fluorescence of particles and fluids were taken by a CCD camera (Grasshopper^®^3, Point Grey Research, Inc.) mounted on the top of a stereo microscope (SZX12, Olympus Corp.) with UV-light capabilities. When more than one size of particles were used in the array, each had a specific fluorescence that resulted in a different intensity that was easily observable. Both white light and fluorescence images of the asymmetric traps were taken both before and after the fluid oscillations (Image J. v.1.49). The row number marks of the white light image were copied, pasted, and aligned on the fluorescence image. Then, the number of particles at each row was counted. In the data analysis for solution exchange experiments, the location of the dispersion front, *r*_*d*_, was the maximum row number with a fluorescence intensity more than two standard deviations above the mean intensity in the pre-mixing non-fluorescent buffer region. The fluorescein concentration in the region of *r*_*d*_ was less than 1% of the concentration of the original fluorescein solution (Supplementary Fig. S4).

### Implementation of fluidic system

LabVIEW was used to control the valve operation and fluid oscillation. The inlets of the control channel were connected to the house pneumatic pressure sources. The LabVIEW-controlled regulators and solenoid valves adjusted the pressure, and the number and amplitude of fluid oscillations, respectively. The amplitude of the fluid oscillation was controlled via adjustment of the duration of solenoid valve opening.

## Supplementary information

Supplementary Information

Movie S1. Segregation

Movie S2. Multiplexed Segregation

Movie S3. Solution Exchange
