# Supplementary material for: Micro-Particle Operations Using Asymmetric Traps
Source: Sci Rep. 2019 Feb 4;9:1278. doi: 10.1038/s41598-018-37454-1 (PMC6362267; doi:10.1038/s41598-018-37454-1)
Supplement: Supplementary file 1 — Supplementary Information [file 41598_2018_37454_MOESM1_ESM.docx]

**Supplementary information**

**Micro-Particle Operations Using Asymmetric Traps**

Jaesung Lee^1^, Sarah E. Mena^1^, and Mark A. Burns^1,2,*^

^1^Department of Chemical Engineering, University of Michigan, Ann Arbor, 48109, USA

^2^Department of Biomedical Engineering, University of Michigan, Ann Arbor, 48109, USA

^*^maburns@umich.edu

**Table S1.** The relative dimensions of the trap gaps and the row shift fraction used for each experiment. d is the particle diameter, h, s and g, are geometrical characteristics of the traps as shown in Figure 1 and ε is the row shift fraction divided by the separation between two adjacent traps. These dimensions can be used to calculate exact coordinates of the points of the array geometry.^1^ *The 29.3 µm is only for the design of the array. The particle diameter for the actual multiplexed segregation experiment was 30.2 µm.

| Experiments | d [µm] | d/h | s/h | g/h | ε |
| --- | --- | --- | --- | --- | --- |
| Binary segregation  /Solution exchange  /Focusing and Splitting | 30.2 | 0.85 | 0.50 | 1.5 | 1/2 |
| Multiplexed segregation  (1^st^ array) | 20.3 | 0.53 | 0.34 | 1.5 | 1/3 |
| Multiplexed segregation  (2^nd^ array) | 29.3^*^ | 0.70 | 0.50 | 1.5 | 1/2 |

**Supplementary Note 1: The particle dynamics in the asymmetric traps and their phase diagrams**

In asymmetric traps, micro-particles typically have one of five different particle dynamics: symmetric passage, symmetric capturing, trap skipping (zig-zag mode), one-way particle transport, or trap skipping (bump mode) (Fig. S1).^1^ Particles in the regime of symmetric passage have a diameter less than the trapping gap of the asymmetric trap, and they freely move back and forth across the array without any trapping. In contrast, particles in the regime of symmetric capturing are trapped in both directions of the oscillatory flow. The trap skipping regime has two different modes: zig- zag and bump. The only difference in these two modes is the way that the particle moves between the traps. The motion of the particle during forward flow is identical for two trap skipping regimes. Finally, as described in the main text, one-way particle transport shows a ratchet-like motion of the particle.


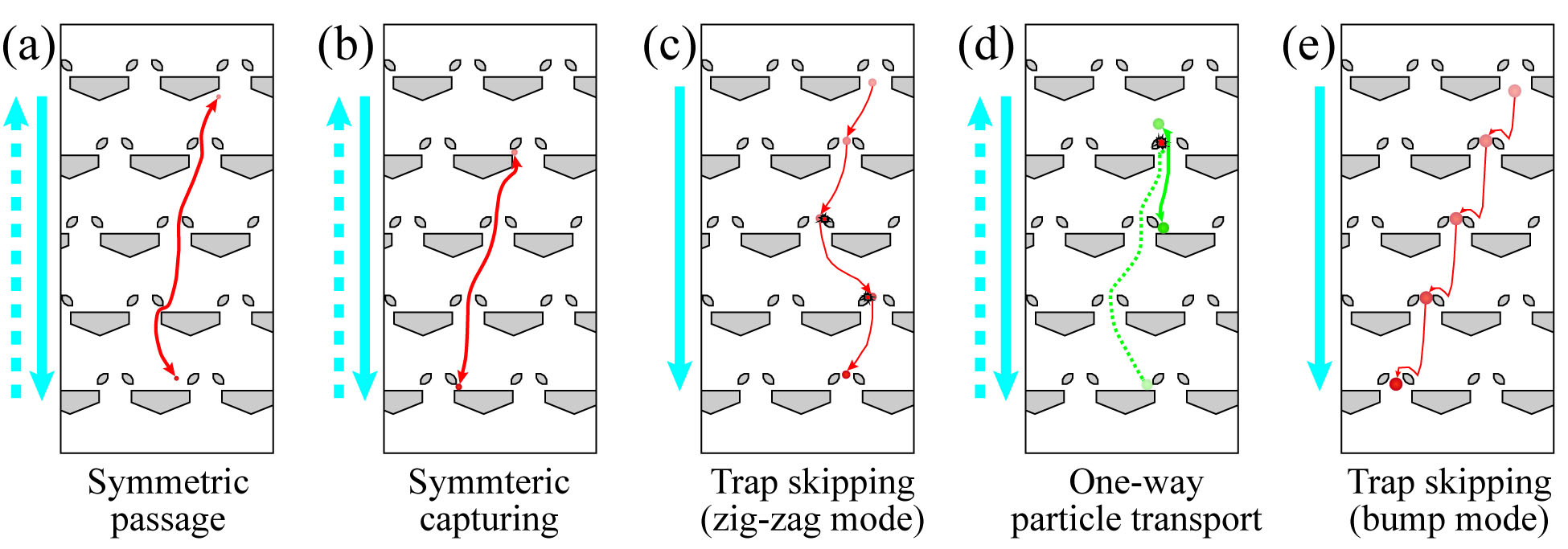


**Figure S1.** Schematic of five types of the particle dynamics in the asymmetric traps: (a) symmetric passage, (b) symmetric capturing, (c) trap skipping (zig-zag mode), (d) one-way particle transport, and (e) trap skipping (bump mode).

The dimensions of the asymmetric traps are determined based on the phase diagram of a specific row shift fraction, ε. From our previous work, we obtained the phase diagrams of four row shift fractions (Fig. S2). For the practical purpose of minimizing the number of trap rows, the row shift fractions of 1/3 and 1/2 are recommended for use.


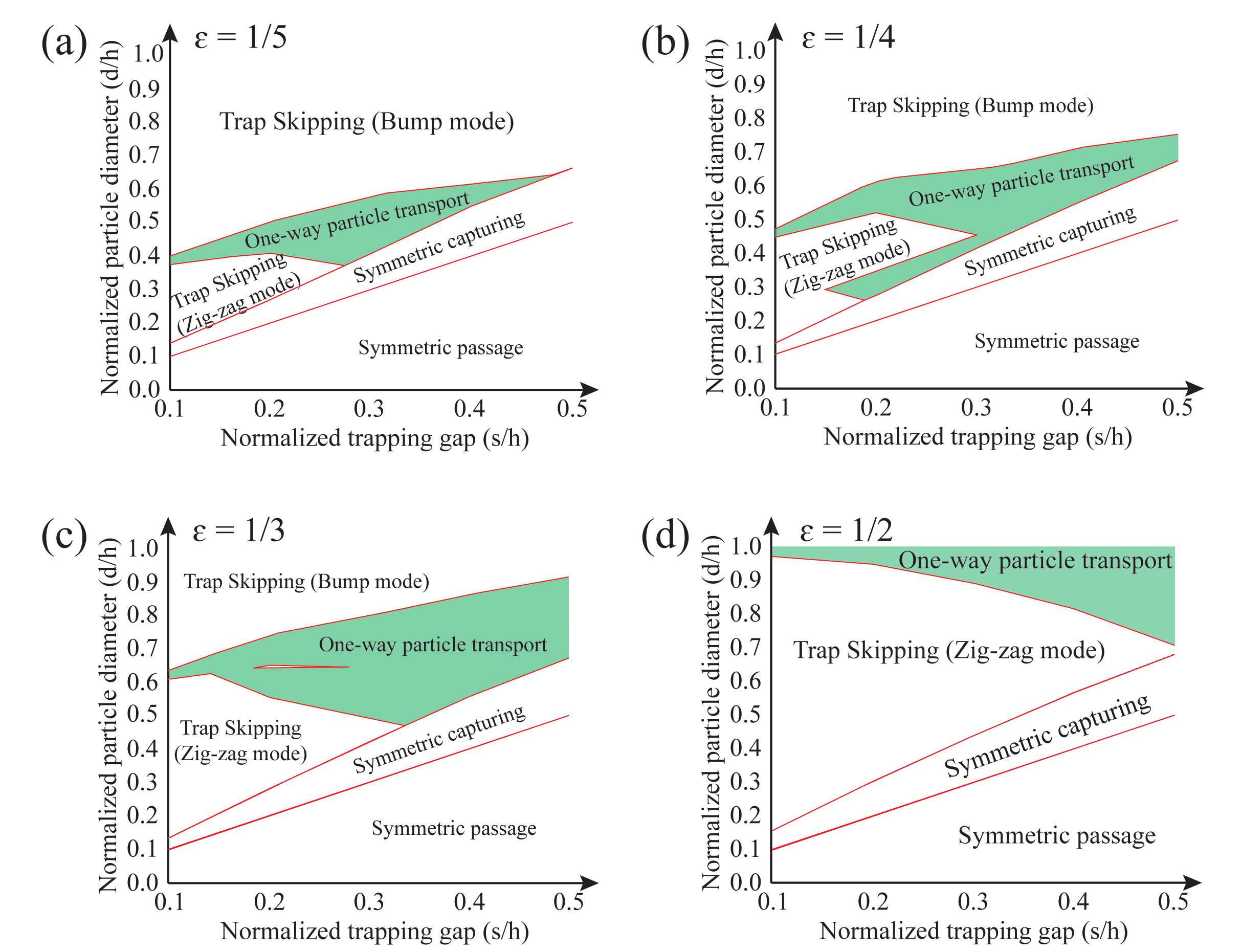


**Figure S2.** The phase diagram of each row shift fraction, ε = (a) 1/5, (b) 1/4, (c) 1/3, and (d) 1/2.

**Table S2.** Graphical description of the variables for quantifying segregation performance. T/t/n represent total, targeted, and non-targeted, respectively. The green (or red) bars show the number of targeted (or non-targeted) particles at each row.


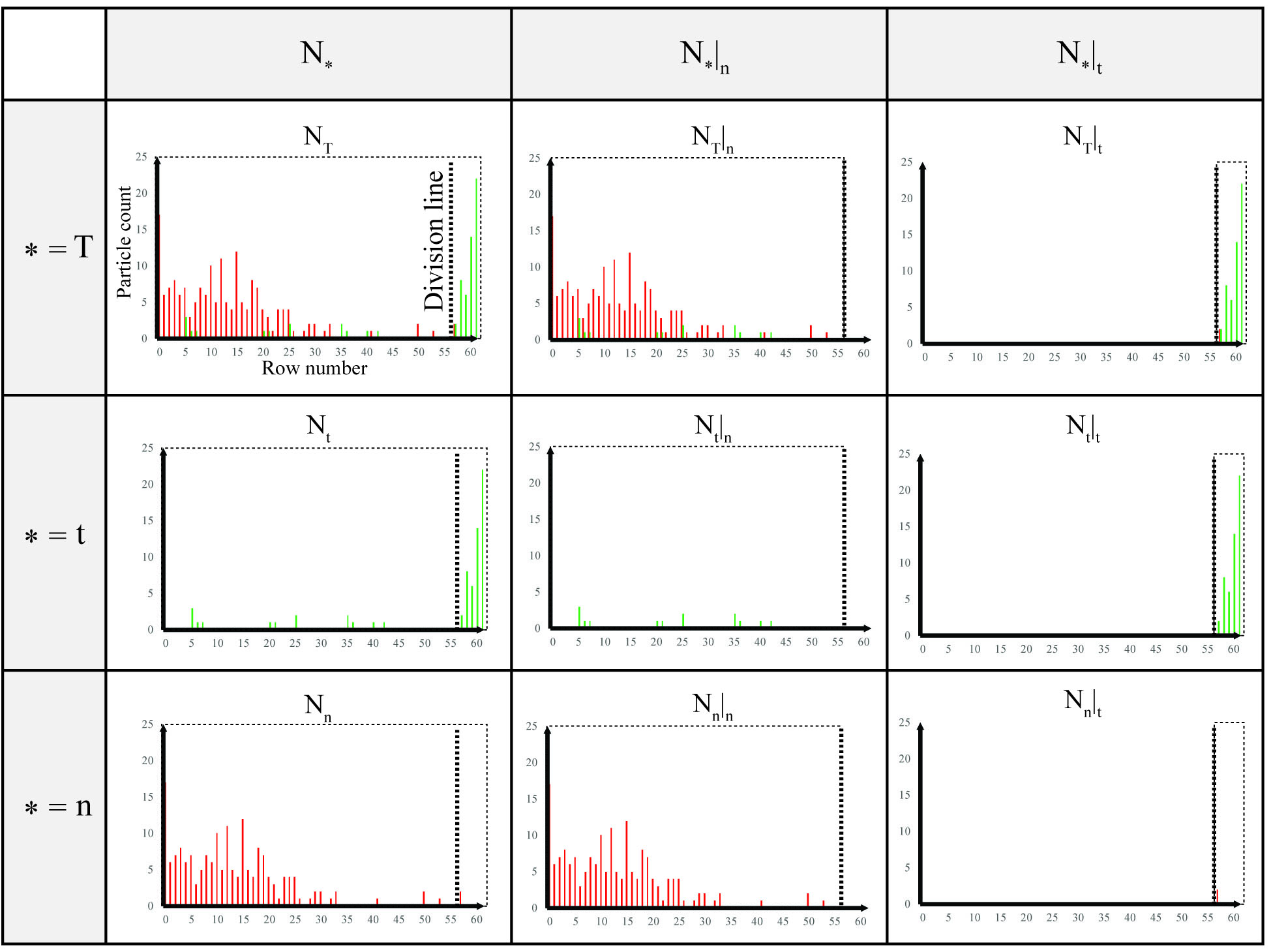


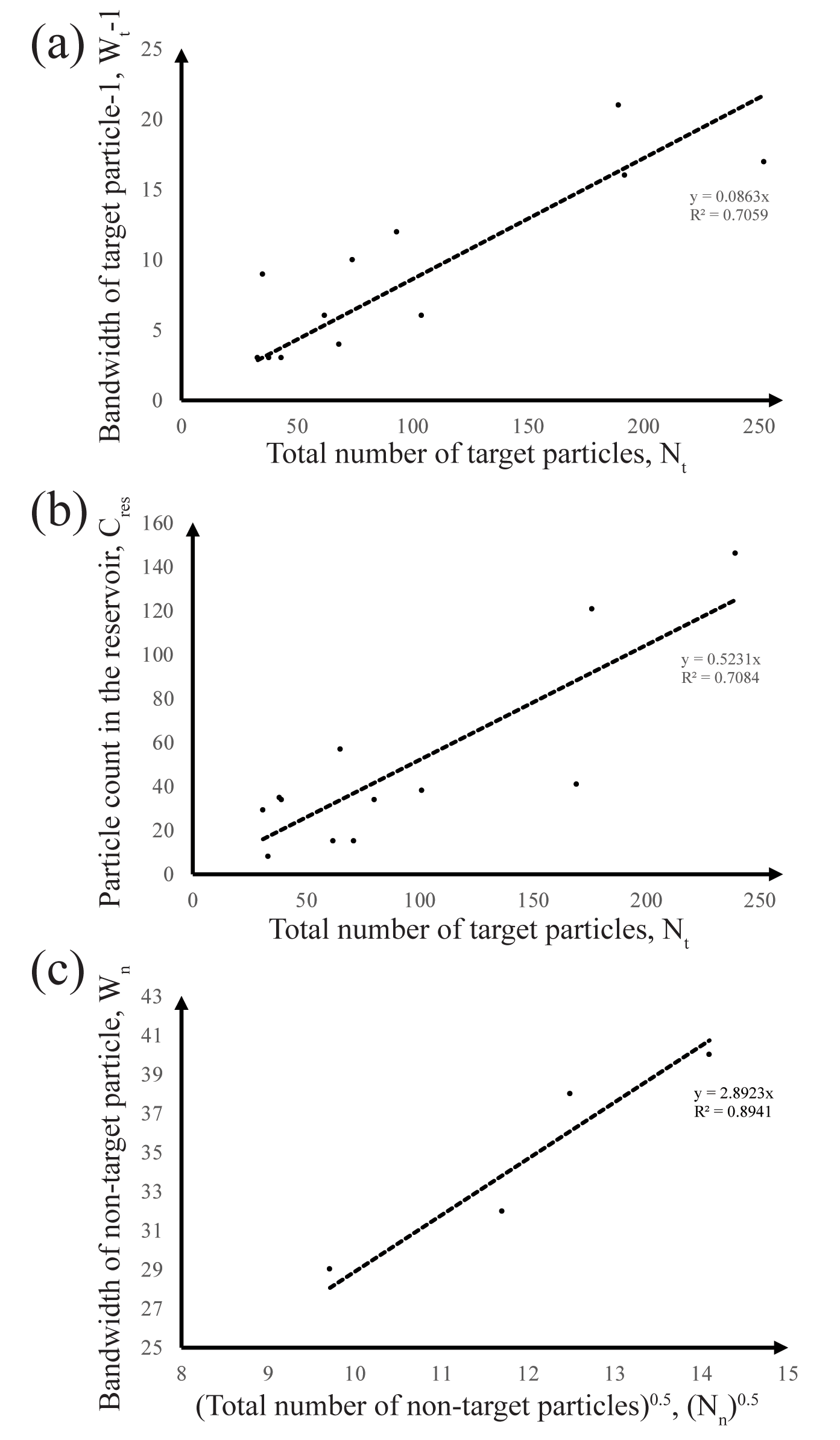


**Figure S3.** The graphs for calculating the bandwidth of particles. (a) Bandwidth vs. total number of target particles in the array excluding the last row. (b) The number of target (30.2 µm) particles accumulated on the last row vs. total number of target particles. We named the last row as the reservoir because the target particles were accumulated at the last row as fluid oscillations progress. Therefore, the bandwidth of target particles is defined as the sum of the bandwidth of target particles in the reservoir and the rest of the array. (c) Bandwidth vs. Total number of non-target (20.4 µm) particles. The bandwidth of non-target particles appears to be linearly proportional to square root of N_n_. This proportionality may originate from the characteristic two-dimensional distribution of the particles in the suspension. In the experiments for the measurement of the bandwidths, a large number and amplitude of oscillations were provided to ensure full transport of the particles.


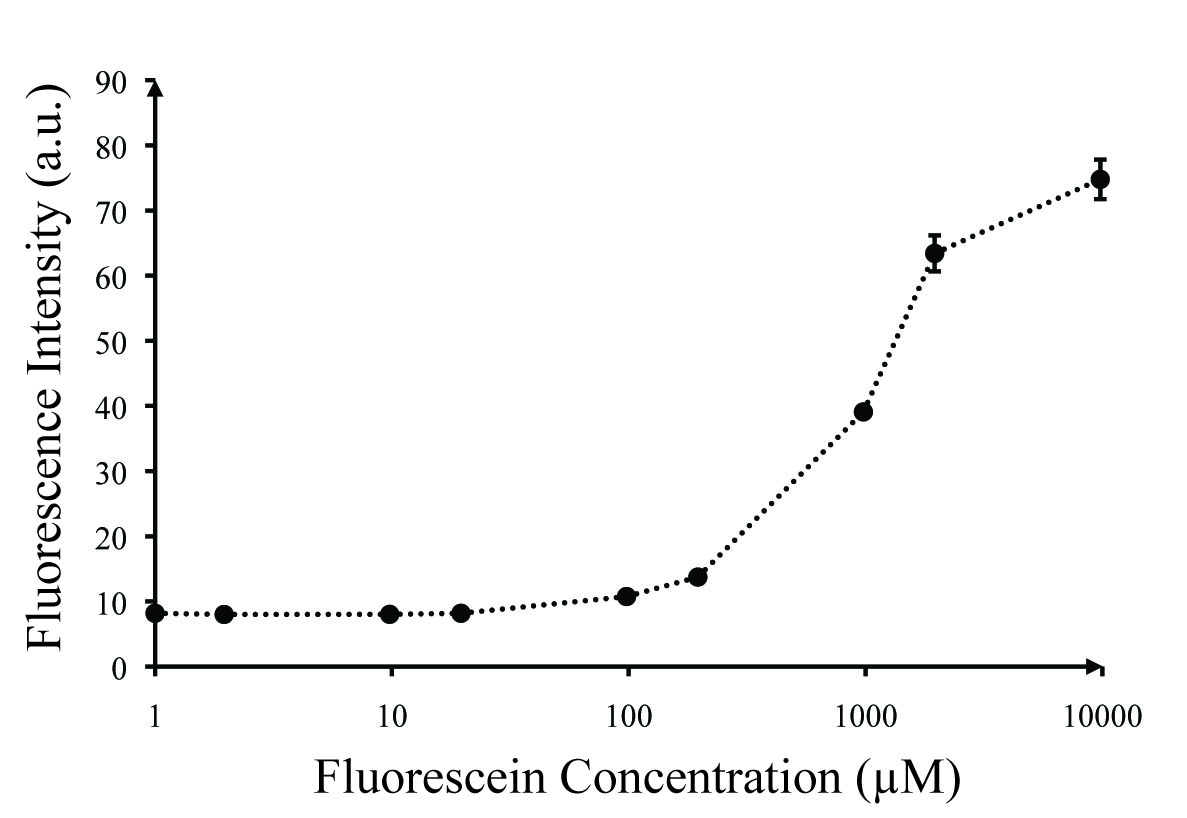


**Figure S4.** A graph of fluorescence intensity vs. fluorescein concentration. For solution exchange experiments, the concentration of the fluorescein solution was about 1.0 x 10^4^ µM. All the r_d_, the boundary of the fluorescein mixing region, had a fluorescence intensity of less than 10.0, which corresponds to a fluorescein concentration less than 100 µM, 1% of the fluorescein solution concentration.

Supplementary Movie Captions

**Movie S1.** Segregation of a binary mixture of micro-particles

30.2 µm particles are transported to the exit of the array of asymmetric traps while 20.4 µm particles are retained in the area near the entry of the array. The transport direction is from left to right. The movie plays 5 times slower than real time.

**Movie S2**. Multiplexed segregation of a ternary mixture of micro-particles

Small particles (10.1 µm) stay in the early part of the first array (left) while large (30.2 µm)- and medium (20.4 µm)-sized particles are transported to the exit of the array. Once the large and medium particles enter the second array (right) at the intermittent large oscillation, only the large particles advance the array. The transport direction is from left to right. The movie plays 5 times slower than real time.

**Movie S3.** Solution exchange of micro-particle

30.2 µm fluorescent particles are transported from fluorescent dye (fluorescein) solution on the left to the other (non-fluorescent) solution. The transport of the particles is faster than the dispersion of the fluorescent dye, resulting in the exchange of the solution surrounding the particles. The transport direction is from left to right. The movie plays at real-time speed.

References

1. Lee, J. & Burns, M. A. One-way particle transport using oscillatory flow in asymmetric traps. *Small* **14**, 1702724 (2018).
